# Sustainability in Health care by Allocating Resources Effectively (SHARE) 5: developing a model for evidence-driven resource allocation in a local healthcare setting

**DOI:** 10.1186/s12913-017-2208-1

**Published:** 2017-05-10

**Authors:** Claire Harris, Kelly Allen, Cara Waller, Sally Green, Richard King, Wayne Ramsey, Cate Kelly, Malar Thiagarajan

**Affiliations:** 1 School of Public Health and Preventive Medicine, Monash University, Victoria, Australia; 2 Centre for Clinical Effectiveness, Monash Health, Victoria, Australia; 3 Medicine Program, Monash Health, Victoria, Australia; 4 Medical Services and Quality, Monash Health, Victoria, Australia; 5 Medical Services, Melbourne Health, Victoria, Australia; 6 Ageing and Aged Care Branch, Department of Health and Human Services, Victoria, Australia

**Keywords:** Health technology, TCP, Disinvestment, Decommission, De-adopt, Resource allocation, Decision-making, Implementation, Model, Framework

## Abstract

**Background:**

This is the fifth in a series of papers reporting Sustainability in Health care by Allocating Resources Effectively (SHARE) in a local healthcare setting. This paper synthesises the findings from Phase One of the SHARE Program and presents a model to be implemented and evaluated in Phase Two. Monash Health, a large healthcare network in Melbourne Australia, sought to establish an organisation-wide systematic evidence-based program for disinvestment. In the absence of guidance from the literature, the Centre for Clinical Effectiveness, an in-house ‘Evidence Based Practice Support Unit’, was asked to explore concepts and practices related to disinvestment, consider the implications for a local health service and identify potential settings and methods for decision-making.

**Methods:**

Mixed methods were used to capture the relevant information. These included literature reviews; online questionnaire, interviews and structured workshops with a range of stakeholders; and consultation with experts in disinvestment, health economics and health program evaluation. Using the principles of evidence-based change, the project team worked with health service staff, consumers and external experts to synthesise the findings from published literature and local research and develop proposals, frameworks and plans.

**Results:**

Multiple influencing factors were extracted from these findings. The implications were both positive and negative and addressed aspects of the internal and external environments, human factors, empirical decision-making, and practical applications. These factors were considered in establishment of the new program; decisions reached through consultation with stakeholders were used to define four program components, their aims and objectives, relationships between components, principles that underpin the program, implementation and evaluation plans, and preconditions for success and sustainability. The components were Systems and processes, Disinvestment projects, Support services, and Program evaluation and research. A model for a systematic approach to evidence-based resource allocation in a local health service was developed.

**Conclusion:**

A robust evidence-based investigation of the research literature and local knowledge with a range of stakeholders resulted in rich information with strong consistent messages. At the completion of Phase One, synthesis of the findings enabled development of frameworks and plans and all preconditions for exploration of the four main aims in Phase Two were met.

**Electronic supplementary material:**

The online version of this article (doi:10.1186/s12913-017-2208-1) contains supplementary material, which is available to authorized users.

## About SHARE

*This is the fifth in a series of papers reporting Sustainability in Health care by Allocating Resources Effectively (SHARE). The SHARE Program is an investigation of concepts, opportunities, methods and implications for evidence-based investment and disinvestment in health technologies and clinical practices in a local healthcare setting. The papers in this series are targeted at clinicians, managers, policy makers, health service researchers and implementation scientists working in this context. This paper synthesises the findings from Phase One of the SHARE Program and presents a model to be implemented and evaluated in Phase Two.*

## Background

Health technologies and clinical practices (TCPs) are defined as therapeutic interventions (including prostheses, implantable devices, vaccines, pharmaceuticals and medical, surgical or other clinical procedures) and diagnostic procedures [1]. Most new TCPs are assessed for safety, effectiveness and cost-effectiveness before they become widespread practice. However there are many longstanding practices that were introduced before rigorous evaluation was required and some recently developed TCPs have been implemented prematurely due to early promise of large benefits, vigorous marketing and patients’ and professionals’ desire for ‘state of the art’ care [2]. As new research emerges it has become clear that some TCPs in current practice do not meet contemporary standards of evidence based care, have been superseded or have become obsolete.

Cessation of TCPs that are potentially harmful, shown to be ineffective, or where a more effective or cost-effective alternative is available has the dual advantage of improving patient care and allowing for a more efficient use of available resources. This concept has become known as ‘disinvestment’. While a lack of common terminology in this area has been noted [3–8], and the multiple definitions for disinvestment are based on different principles [9], the broad concept of removing, reducing or restricting practices that do not work or could be done better or more cheaply is welcome, potentially increasing health benefits without increasing spending.

After implementing a rigorous evidence-based program for assessment of new TCPs prior to their introduction [1], senior leaders at Monash Health (previously Southern Health), a large health service network in Melbourne, Australia, sought to investigate possibilities for a program of disinvestment through the ‘Sustainability in Health care by Allocating Resources Effectively’ (SHARE) Program. The SHARE Program was undertaken by the Centre for Clinical Effectiveness (CCE), an in-house resource to facilitate Evidence Based Practice. An overview of the SHARE Program, a guide to the SHARE publications and further details about Monash Health and CCE are provided in the first paper in this series [2].

The preliminary proposal was for a systematic approach that would integrate systems and processes for transparent, accountable and evidence-based decision-making across the health service. However there is little evidence to inform development of organisation-wide systematic approaches to disinvestment at the local level [7, 10–16].

**Fig. 1:**
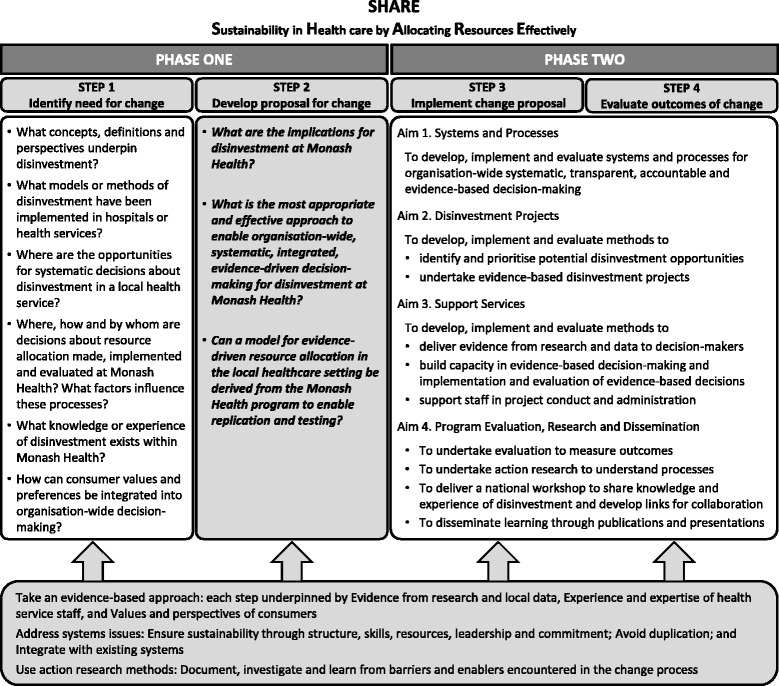
Overview of SHARE Program

In the absence of guidance from the literature, a two-phased process was proposed to identify and then evaluate potential opportunities for disinvestment at Monash Health (Fig. 1). The aim of Phase One was to understand concepts and practices related to disinvestment and the implications for a local health service and, based on this information, to identify potential settings and methods for decision-making. The aim of Phase Two was to implement and evaluate the proposed methods to determine which were sustainable, effective and appropriate at Monash Health.

### Aims

The aim of this project was to develop a proposal for an organisation-wide, systematic, integrated, transparent, evidence-based approach to disinvestment.

The aims of this paper are to outline how the information was collected, synthesised and developed into a proposal for change and to introduce a model of the program to enable replication and testing.

### Research questions

What are the implications for disinvestment at Monash Health?

What is the most appropriate and effective approach to organisation-wide, systematic, integrated, evidence-driven disinvestment at Monash Health?

Can a model for evidence-driven resource allocation in the local healthcare setting be derived from the Monash Health program to enable replication and testing?

## Methods

### Design

#### Model for evidence-based change

The SHARE Program was undertaken using the SEAchange model for Sustainable, Effective and Appropriate evidence-based change in health services [17]. The model involves four steps: identifying the need for change, developing a proposal to meet the need, implementing the proposal and evaluating the extent and impact of the change. Each step is underpinned by the principles of evidence-based practice to ensure that the best available evidence from research and local data, the experience and expertise of health service staff and the values and perspectives of consumers are taken into account. Sustainability, avoidance of duplication and integration of new processes within existing systems are also considered at each step. An action research component enables continuous investigation of the change process to improve the current project and inform future work. The research questions for this paper relate to development of a proposal for change (Fig. 1).

#### Framework for design and evaluation of complex interventions

The two-phased approach taken in SHARE is consistent with the UK Medical Research Council framework for design and evaluation of complex interventions [18]. Phase One involved specifying the context, understanding the problem and defining the components of an optimal intervention. Phase Two was an exploratory trial assessing acceptability and feasibility of the components and identifying methodological issues for implementation and evaluation. These two phases are mapped to the four steps in the model for evidence-based change (Fig. 1).

### Data collection methods and sources

Literature reviews, surveys, interviews and workshops were used to capture the relevant information in Step 1 (Fig. 1). An overview is provided in Table 1 and full details of methods and sources are reported in Additional file 1.

**Table 1:** Summary of data collection methods and sources

Research Question	Method	Source
What are the concepts, definitions and perspectives that underpin disinvestment? [33] What models or methods of disinvestment have been implemented in hospitals or health services? Where are the opportunities for systematic decisions about disinvestment in a health service? [9]	Literature review	Health databases, Internet
	Survey of external experts	Researchers and health librarians interested in disinvestment (15 respondents)
	Semi-structured interviews	Executive of the Technology/Clinical Practice Committee representing Executive Directors, Senior Managers, Clinical Directors (4 members)
	Structured interviews	Key informants purposefully selected to represent Medicine, Surgery, Nursing, Allied Health, Diagnostic Services, Consumers (6 informants)
	Structured workshops	SHARE Steering Committee: Executive Directors, Senior Managers, Clinical Program Directors, Consumers (20 members)
How are decisions about resource allocation currently made at MH? What factors influence decision-making for resource allocation? [1, 29] What knowledge or experience of disinvestment exists within Monash Health? [29]	Structured interviews	Representatives of committees with mandate to make organisation-wide decisions (13 committees); Managers of Approved Purchasing Units (5 managers); Program Directors, Medical Department Heads, Nurse Unit Managers and a Quality Manager in a clinical program with high use of TCPs (9 managers)
	Structured interviews	Representatives of current or completed projects that involved disinvestment-related activities (10 projects)
	Structured workshops	SHARE Steering Committee: Executive Directors, Senior Managers, Clinical Program Directors, Consumers (20 members)
	Structured workshop	Decision-makers from a large multi-campus diagnostic service (18 participants)
	Document analysis	Victorian Department of Human Services and Monash Health documents
How can consumer values and preferences be integrated into organisation-wide decision-making processes? [30]	Literature review	Health databases, Internet
	Semi-structured workshops	Consumer Working Group (3 experienced health service consumer representatives and project team members)
	Structured interviews	Staff responsible for consumer-related activities (2 managers)
	Structured interviews	Representatives of committees with mandate to make organisation-wide decisions (13 committees); Managers of Approved Purchasing Units (5 managers); Program Directors, Medical Department Heads, Nurse Unit Managers and a Quality Manager in a clinical program with high use of TCPs (9 managers)
What do MH decision-makers need to enable access and utilisation of evidence in decision-making? [31]	Literature review	Health databases, Internet
	Structured interviews	Program Directors, Medical Department Heads, Nurse Unit Managers and a Quality Manager in a clinical program with high use of TCPs (9 managers)
	Electronic survey	Clinicians and senior managers representing all sites, clinical programs and professional groups (141 respondents, 103 surveys fully completed)

### Development of proposal for change

#### Project team reflection

An action research approach was adopted based on the ‘researcher as facilitator for change’ model defined by Meyer; researchers working explicitly with and for people rather than undertaking research on them [19, 20]. In this capacity, CCE staff were both the SHARE project team and the action researchers.

CCE staff had regular and ongoing contact over many years with clinicians and managers involved in projects across Monash Health and were familiar with organisational practices, expertise of project staff, available resources, project methods and outcomes. As the SHARE project team, they were able to contribute this knowledge in discussions and decision-making settings.

Observations and reflections of the project team were used for ongoing improvements to the program components and implementation process. An agenda item for ‘Learning’ was scheduled at the beginning of every team meeting. Participants were invited to consider anything that had affected the project since the last meeting using the framework ‘what worked, what didn’t, why and how it could be improved’. Each issue, its effect on the project and potential changes that would build on positive outcomes or remove or minimise future problems were discussed. The learning and actions were documented; actions were assigned, given timeframes and followed up to ensure completion.

#### Analysis and synthesis

Outcomes of consultations and findings from initial interviews with small numbers of participants were simply documented and collated using MS Word or Excel. Workshop and subsequent interview findings were collated in MS Word, Excel and/or Nvivo [21] and analysed thematically by either content analysis [22] to identify emergent themes, or framework analysis [23] when categories had been specified *a priori*. Details of individual project protocols are provided in Additional file 1.

Using the principles of evidence-based change [17], the project team worked with health service staff, consumers and external experts to collate and summarise the findings from published literature and local research and identify the implications for a disinvestment program at Monash Health from the emergent themes.

#### Drafting, review and authorisation of components and activities

Emergent themes were developed into components of the proposed program. Draft proposals, frameworks and plans were developed, reviewed and refined with input from local stakeholders and relevant experts via workshops, presentations and discussions with individuals and groups, consultations and informal discussions (Table 2). Details of structured workshops are provided in Additional file 1, Table E. Decisions were made by the SHARE Steering Committee in workshops held at scheduled committee meetings. Discussion papers and background documents were provided beforehand, formal presentations introduced the workshops, and topics for discussion and decisions required were listed on the agenda. Discussion was informal within the structure of the agenda and decisions were based on consensus. The program was endorsed by the Executive Management Team and Monash Health Board.

**Table 2:** Summary of program development

Objective	Method	Stakeholders and/or Experts
To explore, develop and authorise all program elements, documents and proposals	Structured workshops on specific issues and general discussions at routine meetings	SHARE Steering Committee: Executive Directors, Clinical Program Directors, Senior Managers and Consumers.
To discuss findings of literature review and Consumer Working Group, refine draft consumer participation framework and identify additional issues	Structured workshop	Monash Health Community Advisory Committee
To incorporate feedback from Monash Health leaders	Presentations and discussions with individuals and groups	Individuals: All Medical Program Directors and General Manager of Allied Health; Groups: Nursing Executive
To incorporate feedback from Monash Health staff	Invitation to provide contribution	All staff via the ‘All Staff’ email list; and staff interacting with the project team
To incorporate high level expertise	Consultation	Health Program Evaluator and Health Economist
To determine communication issues and requirements	Consultation	Monash Health Public Affairs and Communication Department
To enhance compatibility and alignment with state health department objectives and funding strategies	Consultation	Victorian Department of Human Services Health Technology Unit
To seek endorsement and support at the highest levels	Presentations and discussions with groups	Executive Management Team; and Monash Health Board

#### Assessment of sustainability

A checklist of factors for success and sustainability, adapted from the work of others [24–27] for use in CCE projects, was used to assess whether there was adequate provision of relevant requirements (structure, skills, resources, commitment and leadership) to achieve and maintain the program components and activities (Table 3) [17].

**Table 3:** Factors for success and sustainability

Success: A proposal is more likely to be successfully implemented if it meets the following criteria. ▪ It is based on sound evidence or expert consensus ▪ It is presented by a credible organisation ▪ It can be tested and adapted ▪ The relative advantage is evident ▪ It is of low complexity ▪ It is compatible with the status quo ▪ It has an attractive and accessible format
Sustainability: A proposal is more likely to be sustainable if it has appropriate and adequate provision in each of the following categories. ▪ Structure ▪ Skills ▪ Resources ▪ Commitment ▪ Leadership

#### Development of a model

Frameworks and models are derived from a set of concepts and the relationships between the concepts to facilitate the development of propositions. The components of the proposed SHARE Program were used as the concepts within the model. Relationships and propositions were derived from the identified needs and a set of sequential processes that emerged from the literature and local findings.

The robustness and usefulness of the proposed model were analysed using the domains outlined for this purpose by Rycroft-Malone and Bucknall [28].

## Results

Results of the literature searches and the response rates and representativeness of participants in surveys, interviews and workshops are included in Additional file 1, Tables A-E. Complete surveys were received from 15 external experts and 118 local respondents, and 90 individuals participated in interviews and workshops. Many participated more than once: as either a representative of more than one role, for example as a committee chair interviewed on one topic and as a clinical department head responding to a survey on another, or to address more than one question, such as a member of the Steering Committee participating in several decision-making workshops.

Data collected from these activities informed a range of research questions. Findings related to research questions not addressed in this paper are reported in other SHARE publications [9, 29–33].

### What are the implications for disinvestment at Monash Health?

Multiple factors for consideration in establishment of the new program were identified. Messages from the literature were consistent with the views of experts and local stakeholders. The findings, sources they were ascertained from, decisions resulting from consultation with stakeholders, and relevant program elements are presented in Table 4. The influencing factors were both positive and negative and addressed aspects of the internal and external environments, human factors, empirical decision-making, and practical applications.

**Table 4:** Factors influencing decisions for program development

Finding	Source	Decision	Program element
Potential benefits of disinvestment identified	Literature	Establish a program exploring disinvestment at Monash Health.	SHARE program
External environment supportive of disinvestment program	Literature & DHS documents		
Internal environment supportive of disinvestment program	Monash Health Staff		
Capacity for leadership in this area demonstrated	Success of new TCP program		
The word ‘disinvestment’ is associated with negative connotations, high risk of engendering suspicion and distrust and getting stakeholders offside.	Literature Monash Health Staff	Proceed carefully, avoid the term ‘disinvestment’ and use positive language.	Principles
‘Top down’ approach seen as negative. Needs to be balanced with ‘bottom up’ strategies and involvement of stakeholders.	Literature Monash Health Staff	Implement ‘top down’ and ‘bottom up’ strategies, make stakeholder engagement a priority, and integrate methods for staff to drive change into the new systems and processes.	Principles
			Preconditions
A systematic integrated approach would be better than ad hoc decisions, individuals ‘championing’ causes or projects undertaken in isolation.	SHARE leaders International experts	Focus on organisation-wide approach to decision-making that integrates new and current systems and processes.	Principles
Perceived lack of transparency and accountability and suboptimal use of evidence in current decision-making processes. Power struggles and hidden agendas perceived to influence outcomes.	Monash Health Staff Project team	Ensure the new systems and processes are transparent, accountable and evidence-based. Introduce explicit criteria for disinvestment decisions.	Principles
Lack of transparency and accountability in reallocation of funding released through disinvestment would be significant barrier to effective program.			
Lack of consistent terminology, absence of decision-making criteria and no guidance to inform an organisational approach.	Literature International experts	Develop our own frameworks and methods.	Principles
Disinvestment should not be considered in isolation but alongside other decisions. Investment and disinvestment decisions are often linked, disinvestment occurs when something new is introduced.	Monash Health Staff SHARE leaders Project team	Do not focus on ‘disinvestment’ or ‘investment’ alone. Consider ‘resource allocation’. Establish processes along decision-making continuum from introduction to removal.	Principles
Health service staff perceive management priorities to be focused on saving money. The concepts around ‘disinvestment’ accentuate this.	Literature Monash Health Staff	Focus on ‘effective application of health resources’ to facilitate a positive approach.	Principles
The program needs a strong positive image that reflects the new focus on ‘effective application of health resources’. Being compatible with ‘iCARE’, the familiar acronym for Monash Health values would be beneficial.	Monash Health Staff SHARE leaders Project team	Change the name from ‘Disinvestment Project’ to ‘SHARE’ (Sustainability in Health care by Allocating Resources Effectively)	Name
Six potential opportunities to integrate disinvestment decisions into organisational infrastructure, systems and processes were identified.	Literature SHARE leaders	Investigate methods to implement disinvestment decisions in the six settings identified.	Systems and Processes
Undertaking disinvestment projects was a key element of the original proposal. Waiting for investigation of the six settings is too long to delay pilot projects. Some ‘quick wins’ would be valuable.	SHARE leaders Monash Health Staff	Develop methods to identify and prioritise potential target TCPs in parallel with the investigation of the six settings. Undertake pilot projects to disinvest them.	Disinvestment projects
Current decisions are made ‘routinely’ or ‘reactively’. Introduction of TCPs is based on applications from clinicians or managers and removal of TCPs is based on emerging problems or product alerts and recalls. Research literature and local data could be used ‘proactively’ to drive health service practice.	Monash Health Staff SHARE leaders Project team	Build on current ‘routine/reactive’ processes that are done well. Develop new processes to use evidence ‘proactively’ to drive decisions and/or priority setting. Make these explicit elements of the new program.	Principles
Using evidence ‘proactively’ requires time and attention from decision-makers. The information provided must be trustworthy, applicable and sufficiently important to warrant adding to their workload.	Monash Health Staff SHARE leaders	Develop methods to identify appropriate high-quality information, process and package it for ease of use and deliver it to the relevant decision-makers.	Systems and Processes
Decisions for resource allocation are delegated to committees and individuals. There are opportunities for improvement in the governance of these processes and to introduce routine consideration of ‘disinvestment’.	Monash Health Staff SHARE leaders Project team	Review processes and governance of decision-making by committees and the authority delegation schedule	Systems and Processes
There is no guidance on consumer participation in disinvestment activities.	Literature	Develop methods to capture and utilise consumer perspectives and integrate them into the new program.	Systems and Processes
With a few exceptions, committees and project teams do not routinely involve consumers in making or implementing decisions and the organisation does not have a framework for engaging consumers.	Monash Health Staff Project team		
The systems and processes for evidence-based decision-making cannot be delivered without appropriate and adequate skills and support	Literature Monash Health Staff	Develop support services that enable capacity-building and provide expertise and practical assistance	Support Services
With a few exceptions, staff do not routinely seek evidence for decisions, are unaware of best practice in implementation and do not evaluate outcomes.	Monash Health Staff Project team	Provide expertise, training and support in accessing and utilising evidence in decisions. Provide expertise, training and support in implementing and evaluating evidence-based change.	Support Services
The main barriers to use of evidence and effective implementation are lack of time, knowledge, skills and resources.	Literature Monash Health Staff		
Health service projects are not usually well supported. It is common for funding to be insufficient, timelines inadequate and staff lacking in knowledge and skills in project management, data collection and analysis.	Monash Health Staff Project team	Influence planning of disinvestment projects to ensure adequate resources and appropriate timelines. Provide expertise, training and support in project methods and administration	Support Services
Disinvestment projects are generally based on health economic principles	Literature	Utilise in-house expertise and take an ‘evidence-driven’, rather than ‘economics-driven’, approach to investigation of disinvestment in the health service context.	Principles
Monash Health does not have expertise in health economics and does not intend to fund this in the foreseeable future	Monash Health Leaders		
Safety, effectiveness, local health service utilisation and benchmarking parameters are possible alternative considerations for disinvestment.	SHARE leaders Monash Health Staff Project team		
Monash Health has high-level expertise in accessing and using research evidence and health service data to inform decisions.			
Monash Health does not have the level of expertise in health program evaluation required for SHARE and has no expertise in health economics.	Project team	Engage consultants in health program evaluation and health economics to assist in development and evaluation	Preconditions
There is no guidance to inform a systematic organisational approach.	Literature	Undertake action research to investigate the process of change in addition to program and economic evaluations. Run a national workshop to learn and share information. Disseminate all findings.	Evaluation and Research
In addition to detailed program and economic evaluation, understanding what happened in the process of investigation, what worked, what didn’t work and why is required.	SHARE leaders Project team		
This large program will need funds. It is consistent with the disinvestment agenda of the Victorian DHS who are sympathetic to a funding application.	DHS documents DHS staff	Seek funding from the state health department.	Preconditions
To be successful this ambitious proposal will need endorsement, support and strategic direction from the highest level and links to those with power and influence in the organisation.	Literature SHARE leaders Project team	Increase membership of the Steering Committee to reflect those best able to provide the appropriate influence, direction and support.	Preconditions
All projects should be aligned to the Monash Health Strategic Goals. Program activities will be facilitated if integrated into the organisation Business Plan.	SHARE leaders Project team	Align SHARE with the Monash Health Strategic Goals and include program activities in the annual Business Plans	Principles

Many of the fundamental decisions in development of the program, such as what to call it and what approach to take, were influenced by both positive and negative factors. For example, respondents felt that the program needed a name that engendered support rather than suspicion and a strong positive image that focused on ‘effective application of health resources’, which was seen as constructive, rather than on disinvestment which was viewed cynically as a strategy to ‘save money’. These findings underpinned the decision to change the name from the ‘Disinvestment Project’ to the ‘Sustainability in Health care by Allocating Resources Effectively’ Program. ‘SHARE’ evoked positive emotions and was compatible with iCARE, a term familiar to all staff as the acronym for the Monash Health values (Integrity, Compassion, Accountability, Respect and Excellence). On a less positive note, respondents perceived significant limitations in organisational decision-making and anticipated that if there was a lack of transparency and accountability in the process of reallocation of resources from disinvestment activities it would be a significant barrier to effective implementation of the program. Based on these findings, transparency and accountability became key principles of the program and all the new systems, processes and decision-making criteria would be made explicit.

Many of the human factors identified are common in health service change initiatives. Although there were a few exceptions, Monash Health staff did not routinely seek evidence for decisions, were generally unaware of best practice in implementation and did not usually evaluate outcomes of decisions. The main barriers to use of evidence and effective implementation and evaluation were lack of time, knowledge, skills and resources. These factors led to proposals for support services to assist staff in making, implementing and evaluating evidence-based decisions.

The lack of information on how to establish organisation-wide systems and processes for disinvestment meant that Monash Health had to rely on empirical reasoning for some decisions. As a result of this approach, two features of the SHARE Program differ significantly from the types of disinvestment activities reported in the literature at the time. Firstly, it was thought that disinvestment should be considered alongside investment in the context of all resource allocation decisions, in contrast to many published examples where it was viewed in isolation. Secondly, a systematic, integrated approach was thought to be better than individual projects that may be driven by ad hoc decisions or individuals ‘championing’ causes. These concepts are reflected in the principles underpinning the SHARE Program.

A number of practical issues were identified across the range of potential activities. Many of these related to factors for success and sustainability of the program such as endorsement, support and strategic direction from the highest level, links to those with power and influence in the organisation, funding, expertise and stakeholder engagement.

### What is the most appropriate and effective approach to organisation-wide, systematic, integrated, evidence-driven disinvestment at Monash Health?

Characteristics of the most appropriate and effective approach for Monash Health were identified from the published literature and local research. These findings underpinned the decisions that defined the program elements (Table 4). These include program components, their aims and objectives, principles that underpin the program, implementation and evaluation plans, and preconditions for success and sustainability.

#### Program components, aims and objectives

##### Systems and processes

Aim 1: To develop, implement and evaluate organisation-wide systematic, transparent, accountable and evidence-based decision-making systems and processes for resource allocation related to health technologies and clinical practices.

**Fig. 2:**
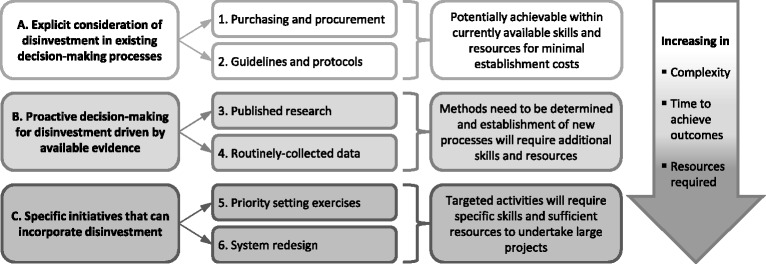
Potential settings for disinvestment (from Harris et al. [9] with permission)

The original aim of the team driving the SHARE initiative was to consider disinvestment in a systematic way, integrating systems and processes for decision-making across the organisation. This was confirmed as the best approach and the earlier aim was refined to replace ‘disinvestment’ with ‘resource allocation’. The proposed objectives involved investigation of six potential settings for decision-making (Fig. 2). Firstly, the nature of the innovations and methods to deliver them would be explored, those thought to be feasible would then be piloted and those found to be sustainable, effective and appropriate would finally be established as ongoing processes.

##### Disinvestment projects

Aim 2: To identify target disinvestment opportunities, establish prioritisation and decision-making processes and develop, implement and evaluate evidence-based disinvestment projects.

It was anticipated that in the longer term the new systems and processes would identify opportunities for disinvestment activities, however the Steering Committee wanted to explore disinvestment projects immediately. This meant that methods to identify and prioritise target TCPs and then implement and evaluate projects to disinvest them must be investigated in parallel to the new organisational systems and processes. These innovations and methods would be explored, piloted and implemented using the approach outlined in Aim 1.

##### Support services

Aim 3: To develop, implement and evaluate support services to provide expertise and facilitate action.

It was clear from the preliminary work that, in order to achieve the first two aims, services to support the proposed activities and build staff capacity and capability would be required. Key areas of need were identified: providing expertise to deliver research evidence and local data to decision-makers, training and supporting staff to use evidence in decision-making and then implement and evaluate their decisions, and training and supporting staff in project methods and administration.

##### Program evaluation and research

Aim 4: To undertake evaluation and research to assess outcomes, understand the process of change and disseminate the findings.

Although each of the first three components included evaluation in the pilot and implementation phases, it was decided to specify a fourth component to highlight the importance of evaluation, research and dissemination in capturing and understanding what happened and sharing this with others interested in developing similar models. Standard health program evaluation methods would be used to assess outcomes, and action research methods would be included to learn about the processes, what worked, what didn’t and why. Running a national workshop was proposed so that the Monash Health team could learn from others with experience in related activities, contribute what had been learned at this point in the SHARE Program, and publish the findings to address some of the gaps in the current literature [34, 35].

#### Principles

A series of principles to underpin the program were identified. These captured the focus of the program (effective application of health resources and decision-making across the continuum from investment to disinvestment), the general approach to program initiatives (evidence-driven decisions and evidence-based development, implementation and evaluation of projects), and specific strategies (routine, reactive and proactive decision-making processes; top-down and bottom-up activities; and alignment with organisational goals and business plans).

#### Preconditions

A number of preconditions were identified to enable this complex multifaceted program to be achieved and maintained. Strategic direction, influence, support and endorsement would be provided by expanding the Steering Committee. Adequate funding was allocated by Monash Health and the Victorian Department of Human Services (DHS). Expertise was sourced in-house and gaps were filled through engagement of expert consultants in health program evaluation and health economics. Organisational readiness for change had been demonstrated and ongoing stakeholder engagement was specified as a priority.

#### Assessment of sustainability

A formal review using the checklist for sustainability developed by CCE was undertaken to assess factors related to structure, skills, resources, commitment and leadership (Table 5). A proposal is more likely to be sustainable if it has appropriate and adequate provision in each category. The SHARE Program met all the requirements adequately.

**Table 5:** Assessment of sustainability

Structure
▪ A Steering Committee is in place with appropriate Terms of Reference and members that can deliver the required strategic direction, influence and support ▪ A Project Team is in place with clear timelines and deliverables ▪ Areas of responsibility are defined and lines of reporting and accountability are clear
Skills
▪ The Steering Committee has expertise in clinical practice, management, finances, operations, legal, ethics, research, information technology, procurement and biomedical engineering ▪ The Project Team has expertise in evidence based practice, knowledge brokerage, implementation and evaluation of change ▪ Additional expertise is available − Collection and analysis of health service utilisation and cost data (Monash Health Clinical Information Management unit) − Program evaluation and health economics (Consultants)
Resources
▪ Appropriate funding has been obtained from Monash Health and Victorian Department of Human Services ▪ Accommodation and infrastructure for project team provided within the Centre for Clinical Effectiveness
Commitment
▪ Monash Health has committed significant funding and program activities are included in the Business Plan ▪ The Board and Executive Management Team have endorsed the program ▪ Three Executive Directors are on the Steering Committee ▪ The Centre for Clinical Effectiveness has prioritised development of organisational infrastructure to support evidence-based practice as a key element in its workplan
Leadership
▪ The same team that developed the award-winning new technology program are leading the SHARE program ▪ Monash Health has expressed a wish to be leaders in disinvestment ▪ The Victorian Department of Human Services has expressed a wish to be leaders in disinvestment ▪ The Centre for Clinical Effectiveness is a leader in enabling evidence-based decision-making ▪ The Steering Committee carries influence (Executive Directors, Program Directors, Senior Management)

#### Implementation

The SHARE Program emerged as a series of projects within each of the four components. Individual implementation plans were developed for each project with strategies based on assessment of barriers and enablers in the relevant context. The interventions were piloted and refined prior to final implementation. These details are published separately [29–32, 36, 37].

The overall program had a general implementation plan couched in terms of timelines and deliverables [38]. Broad consideration of barriers and enablers at the program level was undertaken in the analysis of ‘implications for disinvestment at Monash Health’ and these were addressed in development of the ‘most appropriate model’.

#### Evaluation

A formal Evaluation Framework and Plan was created and included evaluation domains, audience, scope, evaluation questions, sources of data, methods of collection and analysis, reporting and timelines [38]. This was considered to be a dynamic document that could be revised during the program.

An external health program evaluator consulted to the SHARE Program in the role of ‘critical friend’ [38] and a health economist provided expertise and advice for economic evaluations.

A theoretical framework for evaluation of implementation of an evidence-based innovation was used [2] and an outcomes hierarchy based on the SHARE Program components was developed and included in the Evaluation Framework [38].

Due to the size and complexity of SHARE, and its interconnectedness with other Monash Health activities, advice from the SHARE health economist was that an economic evaluation of the overall program would not be possible. Economic evaluation would be limited to the disinvestment pilot projects.

Each of the individual projects in the second phase of SHARE had their own evaluation plans which are reported separately [31, 32, 36].

### Can a model for evidence-driven resource allocation in the local healthcare setting be derived from the SHARE Program to enable replication and testing?

#### Framework

The purpose of a framework is to provide a frame of reference, organise and focus thinking and assist interpretation. Frameworks are descriptive, tend to be high-level and can apply to a wide variety of situations [28, 39].

A framework for SHARE was developed and revised. It was used to clarify thinking; inform purpose, direction and planning; and act as a communication tool.

The initial draft was created to facilitate discussion by the project team and Steering Committee to establish the nature and direction of the program (Table 6). It introduced three main concepts.

- ▪ The original plan for a project about ‘disinvestment’ was reframed to a program addressing the spectrum of decisions from investment to disinvestment across the organisation.
- ▪ There are existing processes at Monash Health for introduction of new TCPs and removal of TCPs in current use, but these are usually ‘reactive’ decisions made in response to internal applications or external notifications.
- ▪ Evidence from published research and local data could be used ‘proactively’ to drive decision-making.

**Table 6:** Initial draft of SHARE framework

Introduction of safe, effective, cost-effective TCPs	Removal of harmful, ineffective, inefficient TCPs
Reactive (current) • Application process Proactive (potential) • Identification of evidence regarding new TCPs that are safer, more effective or more cost-effective	Reactive (current) • Drug alerts, product withdrawals Proactive (potential) • Identification of evidence regarding TCPs in current practice that are less safe, less effective or less cost-effective

**Fig. 3:**
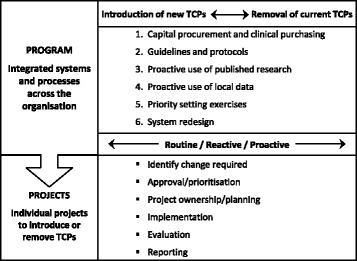
Model for exploring Sustainability in Health care by Allocating Resources Effectively in the local healthcare setting

A revised draft was developed to reflect subsequent decisions and was used for presentations to the Executive Management Team and the Board to seek endorsement and to the Victorian DHS for funding (Fig. 3). It retained the key concepts noted above and introduced another two.

- ▪The six settings identified as potential opportunities for decision-making in a systematic, integrated organisation-wide program should be explored across the continuum from investment to disinvestment and should address routine, reactive and proactive decision-making processes.
- ▪ A ‘program’ of integrated systems and processes identifying TCPs for introduction, restriction or removal would initiate and direct a series of methodologically rigorous ‘projects’ implementing the desired changes.

#### Model

A model is more precise and more prescriptive than a framework. It is narrower in scope, the concepts are well defined and the relationships between them are specific. Models are representations of the real thing [28, 39].

**Fig. 4:**
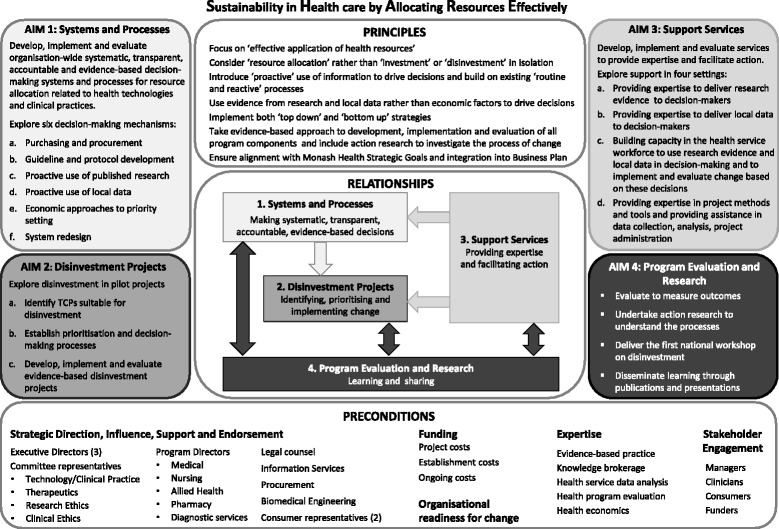
Revised draft of SHARE framework

The final representation of the SHARE Program, created for this paper, captures all the program elements and their relationships (Fig. 4). It is precise, prescriptive and provides sufficient detail to be a model for a systematic approach to evidence-based resource allocation in a local health service.

##### Concepts

The components of the proposed SHARE Program (aims and objectives, underpinning principles, preconditions for success and sustainability) are the concepts within the model.

##### Relationships

The initial proposal had two aims, to develop systems and processes for decision-making and to undertake disinvestment projects. The systems and processes would lead to identification of target TCPs to be disinvested in individual projects. This sequential process is represented by an arrow from Aim 1 to Aim 2.

Based on information from the literature and stakeholder feedback it was clear that these two aims would not be successful without provision of expertise and support to facilitate decision-making (systems and processes) and implementation of change (projects). These needs are represented by arrows from Aim 3 to Aims 1 and 2.

Detailed program evaluation and research to measure and understand the change process were considered to be a vital fourth component and would be applied to the other three components. The double headed arrows between Aim 4 and Aims 1, 2 and 3 indicate that evaluation and research inform further development of the components which in turn would be evaluated and researched.

The Principles and Preconditions sit above and below the four aims indicating that they apply to the whole program.

##### Propositions

A series of propositions can be derived from the components and their relationships.

- ▪ Systems and processes will be required for systematic, integrated, transparent, accountable, evidence-based decision-making in an organisation-wide approach to identification of potential disinvestment opportunities.
- ▪ Projects arising from these decisions will be undertaken to confirm potential benefits, harms and the priorities for disinvestment of identified targets, and implement and evaluate disinvestment where appropriate.
- ▪ Support services that provide expertise, training and support to decision-makers and project staff in finding and using evidence from research and local data in decision-making, implementation, evaluation and project management will be required for the systems, processes and projects to be successful.
- ▪ Evaluation and research of the systems, processes, projects and support services will inform and enable quality improvement, organisational learning and development, and will add to the body of knowledge on disinvestment.

##### Characteristics of the model

The model is primarily descriptive to enable application in a local healthcare service and allow replication and testing. It was developed using both deductive and inductive methods. Although not based on a specific theory, it has potential to facilitate future theory development and/or testing. Specific characteristics of the model and potential for its use, as discussed in the sections above, are summarised in Table 7 using domains and criteria developed to assess the robustness and utility of proposed models and frameworks [28]. This overview enables potential users to identify whether the model will meet their aims and be applicable to their situation.

**Table 7:** Features of a model for systematic approach to resource allocation in a local health service

Domain	SHARE features
Purpose ▪ descriptive, explanatory or predictive	The model is primarily descriptive to enable replication and testing in other settings. There are also some explanatory elements addressed in the relationships between components, for example the systems, processes and projects are thought to require input from the four support services to ensure successful implementation
Development ▪ deductive or inductive ▪ supporting evidence	Methods used in development were both deductive and inductive. Evidence from the research literature and other publications, health service staff, consumers and external experts was used.
Theoretical underpinning ▪ explicit or implicit	No specific theory was used to underpin the model. A theoretical framework for evaluation of implementation of an evidence-based innovation was used to design questionnaires for data collection to inform program development.
Conceptual clarity ▪ well-described, coherent language for identification of elements ▪ strengths and weaknesses of theories ▪ potential to stimulate new theoretical developments	Four components are outlined in the model. The relationships between them are delineated in a simple diagram. The details of each component, the aims and objectives, are provided in the surrounding boxes. The principles that underpin the program and the preconditions for success and sustainability are also detailed in surrounding boxes. No specific theories were used so no comparisons are made. There is potential for new theoretical developments if: ▪ specific theories are tested in development and implementation of the components ▪ components are removed or the relationships changed ▪ principles or preconditions are varied ▪ the model is applied in contexts other than resource allocation for technologies and clinical practices ▪ the model is applied in settings other than local health service networks
Level ▪ individual, team, unit, organisation, policy	The program was developed for organisation-wide implementation in a local health service. This approach could also be used at a higher (regional, state/provincial, national) or lower (single facility, department or unit) level, however is unlikely to be applicable to individual decision-makers.
Situation ▪ hypothetical, real	The model represents actual settings and contexts in health service decision-making and implementation of change. However it could also be used for teaching or capacity building through hypothetical classroom discussions or simulation exercises.
Users ▪ nursing, medical, allied health, policy makers, multidisciplinary	SHARE focuses on decision-makers at the organisation-wide level in a local health service. This includes senior clinicians, managers and policy makers across all professional disciplines, all clinical settings and some areas of corporate practice (eg finance, procurement, legal, ethics, IT, biomedical engineering); and health service consumers.
Function ▪ barrier analysis ▪ intervention development ▪ selection of outcome measures ▪ process evaluation	The main function is to enable replication and testing of the SHARE program by capturing the components and their relationships, principles and preconditions. The principle of an evidence-based approach to change requires assessment of barriers and enablers but the model itself does not specifically facilitate this process. The model would assist in development of an intervention for systematic evidence-based decision-making and implementation of change. Evaluation of process and outcomes is a key element, however selection of variables and outcome measures is not facilitated by the model per se, but an evaluation framework and plan has been developed [38].
Testable ▪ hypothesis generation ▪ supported by empirical data ▪ suitable for different methodologies	The model describes settings and opportunities, systems and processes, and structures to support decision-making, implementation of change and evaluation of process and outcomes. A range of hypotheses could be developed for each of these elements and the relationships between them which could be tested in a number of ways using various methodologies.

## Discussion

### Strengths

The main strengths of this process arise from the evidence-based and explicit approach. Decisions were based on information from the research literature and local data collected for this purpose, integrated with the views of experts in the field and local health service staff and consumers. This approach facilitates development of strategies that are more likely to be sustainable, effective and appropriate [17, 40]. The broad stakeholder involvement enables local ownership and the transparency of the process leads to trust.

A rigorous evidence-based approach was possible due to the provision of adequate resources. CCE staff had appropriate skills for this work and adequate time was allocated to undertake it.

The timing of the project was opportune as internal and external environments were both amenable to exploration of disinvestment. The international literature on methods of disinvesting individual TCPs was building, the Victorian DHS was exploring the role of disinvestment at state level and all the staff and consumers approached were constructive in their responses. Monash Health had already demonstrated commitment and leadership to evidence-based decision-making with the new Technology/Clinical Practice Program [1]. The preliminary work for SHARE was able to capitalise on this momentum.

Staff and consumers were in agreement in their responses. Themes regarding current practice, proposals for change and barriers and enablers were strong and consistent across all participant groups.

The key messages arising from local responses were consistent with the literature at the time and remain consistent with current publications [41, 42].

One aspect of the proposed model initially appeared to be a limitation, but when considered in light of the current literature may be seen as a strength. The available research in disinvestment was predominantly focused in health economics but Monash Health had no expertise in this area and did not intend to employ a health economist. The decision to take an ‘evidence-driven’ approach to disinvestment was based on the available in-house expertise. The proposed ‘evidence-driven’ model is novel and untested, however there are some encouraging findings in more recent research that indicate it might be well-suited to health service decision-making. Access to robust evidence, rather than an emphasis on cost saving, is thought to improve disinvestment decision-making and disinvestment is reported as more likely to be accepted by both clinicians and consumers if the focus is on quality and safety [40, 43]. Surveys indicate that most decision-makers in the health sector do not routinely use economic evaluations in their decisions [14, 44]. Two large international surveys on use of evidence in decision-making have been conducted recently. Ninety-nine per cent of respondents from 15 countries indicated that systematic consideration of the available evidence would improve health system decision-making [45]. The second survey found that clinicians and policy-makers from 23 countries considered clinical effectiveness, safety, quality of evidence, disease severity and impact on healthcare costs to be the most relevant criteria [46]. A systematic review of decision criteria for resource allocation summarised the frequency of criteria cited by 40 studies: equity/fairness (*n* = 32), efficacy/effectiveness (*n* = 29), stakeholder interests and pressures (*n* = 28), cost-effectiveness (*n* = 23), strength of evidence (*n* = 20), safety (*n* = 19), mission and mandate of health system (*n* = 19), organizational requirements and capacity (*n* = 17), patient-reported outcomes (*n* = 17) and need (*n* = 16) [47]. The proposed Monash Health ‘evidence-driven’ model and the suite of criteria used in the Technology/Clinical Practice Program [1] capture all these criteria so is likely to be compatible with current attitudes and behaviours of decision-makers.

### Limitations

As there was no guidance on how to approach disinvestment from an organisation-wide perspective, the SHARE model was developed de novo by integrating theoretical and generic principles with staff and consumer experiences and perspectives. There is still a lack of information related to most of the strategies in the SHARE proposal and a number of recent systematic reviews and discussion papers on disinvestment call for research in these areas [4–7, 11–15, 40, 48–56].

The only clear advice was to avoid the term ‘disinvestment’ due to the negative connotations and lack of common understanding. Although the name and general approach of SHARE was positive and steered away from the concept of ‘disinvestment’, a more appropriate term to describe the ‘Disinvestment Projects’ in Aim 2 proved elusive.

SHARE is about systems and processes in a health service, a complex dynamic organisation with a myriad of context-specific factors. The external validity of the proposed model and generalisability of the SHARE outcomes may be limited as a result. Health services in developed countries are very similar in many ways, but quite diverse in others. The diversity often lies in funding models and organisational culture, both of which may have a considerable impact on decision-making systems and processes for resource allocation. Health services in developing and resource-poor settings may be different in many additional ways that significantly reduce the applicability of findings from an Australian program. Other context-specific factors at local, regional or national level likely to affect generalisability are strategic direction, priorities, infrastructure, available project funding and leadership.

A wide group of stakeholders were engaged to represent consumers and all professional groups, at all relevant levels of seniority, across all campuses. Their responses were overwhelmingly similar with messages that were strong and consistent, which adds confidence to the validity of the process. However it should be kept in mind that those who agreed to participate are potentially more sympathetic to the ideas proposed and may not represent all views.

The project team responsible for delivering the SHARE Program at Monash Health were also the researchers investigating the processes undertaken. This has the potential to introduce subjectivity into the evaluations and limit insight if organisational assumptions are accepted without challenge. Extensive stakeholder involvement, transparency of methods and participation of an external evaluator in the role of ‘critical friend’ [38] were included in the SHARE processes to minimise these limitations.

The SHARE model utilises the in-house expertise of staff in evidence-based practice, knowledge brokerage and data analysis. Health services that do not have high-level skills in these areas may not be able to replicate this model without adaptation. The systematic approach could still be undertaken but with alternative drivers for change. Those with access to health economists in-house or in partnership with a local university could focus on economic principles, an option not available to Monash Health. Those without access to expertise in health economics or evidence-based decision-making may develop other methods such as a consensus-driven approach.

### Implications for policy and practice

Local research identified a number of weaknesses in organisational decision-making such as lack of explicit criteria; limited use of evidence; staff under-skilled and under-resourced to make, implement and evaluate evidence-based decisions; and minimal consumer involvement [29, 31]. Monash Health is not unique and these issues are commonplace in health services around the world [8, 40, 44, 51, 57–60]. This indicates enormous opportunities for improvement through strategies that address these limitations.

The importance of allowing adequate time for development, implementation and evaluation of innovations is well established. Yet this is a constant tension in health services where a common response to an emerging problem is often urgent and reactive, delivered by staff with no experience in project management or change strategies, with inadequate resources and inappropriate timelines, which is not implemented or evaluated effectively [29, 40]. It is anticipated that a systematic integrated approach to organisational decision-making for resource allocation will require considerable time, skills, resources and support, all of which are traditional challenges for health services, however once established the model will allow more timely reaction to future challenges.

### Implications for research

SHARE is a suite of integrated initiatives to improve health service decision-making. Although the evaluation design is rigorous and an action research process is built around the program components to understand the process of change, the primary objective is quality improvement rather than research. As an exploratory study in the UK Medical Research Council framework for developing complex interventions [18], SHARE illustrates concepts, issues, barriers and enablers to evidence-based disinvestment in a local health service. These findings can be tested in controlled studies in a range of contexts to enable recommendations for effective practice.

The findings and decisions that underpinned program development are outlined (Table 4) and the model describes settings and opportunities, systems and processes, and structures to support decision-making, implementation of change, and evaluation of process and outcomes (Fig. 4). These details will enable replication of the program, testing of assumptions and comparison of characteristics of the environment, stakeholders and intervention.

There is potential for new theoretical developments if, for example:

- ▪ specific theories are tested in development and implementation of the components
- ▪ components are removed or the relationships changed
- ▪ principles or preconditions are varied
- ▪ the model is applied in contexts other than resource allocation for TCPs
- ▪ the model is applied in settings other than local health service networks

## Conclusion

A robust evidence-based investigation of the research literature and local knowledge with a range of stakeholders resulted in rich information with strong consistent messages. The process was made possible by provision of appropriate resources, expertise, time and support. The implications for disinvestment in the local healthcare setting were many and varied. The influencing factors were both positive and negative and addressed aspects of the internal and external environments, human factors, empirical decision-making, and practical applications. At the completion of Phase One, synthesis of the findings enabled development of frameworks and plans, and all preconditions for exploration of the four main aims in Phase Two were met. The model for sustainability in health care by allocating resources effectively can be replicated or adapted by health services wishing to establish a program for disinvestment and tested by researchers to confirm, refute or understand the processes involved.

## Additional file

Additional file 1: Details of methods (PDF 366 kb)

## Abbreviations

CCE: Centre for Clinical Effectiveness
DHS: Department of Human Services
iCARE: Compassion, Accountability, Respect, Excellence (Monash Health values)
SHARE: Sustainability in Health care by Allocating Resources Effectively
TCPs: Technologies and clinical practices
