# Erratum to: Sustainability in Health care by Allocating Resources Effectively (SHARE) 5: developing a model for evidence-driven resource allocation in a local healthcare setting

**DOI:** 10.1186/s12913-017-2443-5

**Published:** 2017-08-30

**Authors:** Claire Harris, Kelly Allen, Cara Waller, Sally Green, Richard King, Wayne Ramsey, Cate Kelly, Malar Thiagarajan

**Affiliations:** 1 School of Public Health and Preventive Medicine, Monash University, Melbourne, VIC Australia; 2 Centre for Clinical Effectiveness, Monash Health, Melbourne, VIC Australia; 3 Medicine Program, Monash Health, Melbourne, VIC Australia; 4 Medical Services and Quality, Monash Health, Melbourne, VIC Australia; 5 Medical Services, Melbourne Health, Melbourne, VIC Australia; 6 Ageing and Aged Care Branch, Department of Health and Human Services, Melbourne, VIC Australia

## Erratum

The captions for Figures 3 and 4 were switched during the production process. BMC HSR apologises for this error. The caption for Figure 3 should read ‘Revised draft of SHARE framework’. The caption for Figure 4 should read ‘Model for exploring Sustainability in Health care by Allocating Resources Effectively in the local healthcare setting’.
