# Supplementary material for: Sustainability in Health care by Allocating Resources Effectively (SHARE) 5: developing a model for evidence-driven resource allocation in a local healthcare setting
Source: BMC Health Serv Res. 2017 May 10;17:342. doi: 10.1186/s12913-017-2208-1 (PMC5424307; doi:10.1186/s12913-017-2208-1)
Supplement: Additional file 1: — Details of methods (PDF 366 kb) [file 12913_2017_2208_MOESM1_ESM.pdf]

Sustainability in Health care by Allocating Resources Effectively (SHARE) 5: Developing a model for evidence-driven resource allocation in a local healthcare setting

Additional File: Methods

Contents

Table A 2

What are the concepts, definitions and perspectives that underpin disinvestment? 2

What models or methods of disinvestment have been implemented in hospitals or health services? 2

Where are the opportunities for systematic decisions about disinvestment in a health service? 2

Table B 4

How are decisions about resource allocation currently made at Monash Health? 4

What factors influence decision-making for resource allocation? 4

What knowledge or experience of disinvestment exists within Monash Health? 4

Table C 6

How can consumer values and preferences be integrated into organisation-wide decision-making processes? 6

Table D 8

What do Monash Health decision-makers need to enable access and utilisation of evidence in decision-making? 8

Table E 9

Program development 9

References 10

Table A

What are the concepts, definitions and perspectives that underpin disinvestment?

What models or methods of disinvestment have been implemented in hospitals or health services?

Where are the opportunities for systematic decisions about disinvestment in a health service?

Reproduced with permission from SHARE Paper 2 [1]

**Literature review**
**Aim:** To understand the concepts related to disinvestment and their implications in a local health service and to ascertain examples of existing decision-making systems and processes in this setting.<br>**Search terms:** Medical Subject Headings (Health Care Rationing, Resource Allocation, Health Priorities and Health Services Needs and Demand) and Text words (disinvestment, decommissioning, defunding, resource release, allocation, reallocation, hit list, ineffective services, low value services, wish list, exclusions, priority setting, program budget marginal analysis, PBMA, resource scarcity, rationing, invest to save) were used with truncations appropriate to the databases utilised. The search strategy was iterative with new terms added as they were identified.<br>**Sources:** Medical databases (Ovid Medline, All EBM Reviews, EMBASE, Cochrane Library), the internet (via the Google search engine) and guideline websites. These methods were supplemented by follow up of reference lists in key publications and searches for publications by identified authors in the field.<br>**Inclusion criteria:** English language publications including guidelines, reviews, research studies, technical reports or policy documents that addressed the issue of disinvestment from a conceptual (terminology, definitions and operational criteria) or policy perspective.<br>**Data Collection and Analysis:** Inclusion, exclusion and appraisal criteria were established a priori. Publications that did not meet the criteria were excluded on review of title and abstract. When a decision could not be made based on abstract alone, full text was retrieved. Critical appraisal appropriate to study design was planned but no research studies were identified.<br>**Search results:** Nineteen documents met the inclusion criteria. These were mainly publications providing a statement of the policy context, the rationale or need for disinvestment and/or a critique of existing processes. A small number of case reports were included but no research studies were identified.<br>**Synthesis:** Information from articles which met the inclusion criteria was summarised based on content relevant to the themes of conceptual and policy perspectives determined a priori.<br>Full details are in the review publication [2].
**Interviews with members of the Technology/Clinical Practice Committee**
**Aim:** To identify opportunities for disinvestment in existing or potential decision-making settings and consider implications for disinvestment in the Monash Health setting<br>**Participants:** The Executive of the Technology/Clinical Practice Committee (TCPC), the initiators of the SHARE Program, included an executive director, medical director, clinical program director and research director.<br>**Data collection:** Semi-structured group and individual discussions were conducted using prompts based on the two aims; discussions were documented in the minutes.<br>**Collation:** Responses were collated and added to findings from the other sources which were then analysed thematically by content analysis.<br>**Response rate:** All 4 informants participated.<br>**Representativeness of sample:** Participants represented senior decision-makers from a range of contexts
**Survey of external experts**
**Aim:** To ascertain unpublished experiences or examples of models or methods for disinvestment in the local healthcare setting.<br>**Participants:** 1) Disinvestment researchers initially identified from publications and websites about disinvestment and subsequently using a snowballing technique based on feedback from respondents. 2) Subscribers to the Health Technology Assessment (HTA) email list.<br>**Design:** The organisation-wide systematic approach to disinvestment proposed in the SHARE Program was described in an email. Participants were asked if they had experiences of disinvestment in the local healthcare context that could inform Monash Health decision-making, any unpublished reports or other documents on this topic, and current or planned research in this area.<br>**Data collection:** Responses were received by return email.<br>**Analysis:** Responses were collated and added to findings from the other sources which were then analysed thematically by content analysis.<br>**Response rate:** Eleven of the 14 researchers and four health librarians from the HTA list (denominator unknown) responded to the survey.

**Structured workshops with the SHARE Steering Committee**
**Aim:** The workshops had several aims, the component reported in this paper relates to identification of opportunities for disinvestment in existing or potential decision-making settings.<br>**Inclusion criteria:** Senior decision-makers at Executive and Director level and health service consumers<br>**Sampling:** Convenience sampling was used to include members of the SHARE Steering Committee comprising Executive Directors (Medical, Nursing, Support Services), clinical Program Directors (Medical, Nursing, Allied Health, Pharmacy, Diagnostic Services), Committee chairs (Technology/Clinical Practice, Therapeutics, Human Research and Ethics, Clinical Ethics), Directors of non-clinical services (Information Services, Clinical Information Services, Procurement, Biomedical Engineering, Research Services), Legal counsel and two consumer representatives. Two representatives from the Department of Human Services Technology Division also participated.<br>**Approach:** Workshops were conducted at scheduled Steering Committee meetings.<br>**Design:** Workshops were based on the first two steps in the SEAchange model for evidence-based change [3]; identifying the need for change and developing a proposal for change. Presentations outlining the background and aims of the workshops were made by the project team, discussion was structured around the questions to be addressed and decisions were based on consensus. Questions included:<br>*Workshop 1:* Where and how are decisions made, documented, communicated, implemented and evaluated and what are the related system issues? Where is change required? Why? What is the problem? How can the need for change be measured? What are the factors enabling sustainability of the current system? How is it integrated?<br>*Workshop 2:* What existing systems/processes work well that we could maintain as they are, should be ceased, could be kept but require improvement? What new systems/processes should be introduced? What structures, skills, resources, commitment and leadership are required? Are they available? If not, how can they be obtained? What existing systems can be utilised? What is the solution to the problem? What are the options? What is known about best practice in this area? What is required to ensure sustainability of the proposed system? How can it be integrated?<br>**Data collection:** Participants completed prepared worksheets and discussed the findings. Discussion and decisions were documented in minutes.<br>**Respondent validation:** Minutes were approved at the following meeting.<br>**Analysis:** Data from the worksheets and findings from the discussion were collated and organised in MS Word and Excel. Emergent themes were identified by framework analysis.<br>**Response rate:** Thirteen members participated, 9 attended the first workshop, 11 attended the second, and some non-attenders also completed the worksheets.<br>**Representativeness of sample:** A range of senior decision-makers were represented at each workshop, plus representatives from the state health department.
**Interviews with key local informants**
**Aim:** To test preliminary thoughts regarding direction of the SHARE Program with front line staff and consumers<br>**Participants:** Six participants selected purposefully and pragmatically to seek the views of a range of Monash Health decision-makers: the five senior clinicians were program directors and department heads representing medicine, surgery, nursing, allied health and diagnostic services and the consumer representative had experience on committees that made organisation-wide decisions.<br>**Interview schedule**<br>*Disinvestment*: Have you heard about the concept of disinvestment?<br>*Potential settings/methods*: Are you aware of any of these? Do you do any of these sorts of things? What could you do in your Unit? What could be done in your Program/Division? What could be done by your colleagues eg referrers? Any opportunities for quick wins? Incentives to change? Barriers to change? Potential to link into advanced trainee projects?<br>*Research evidence*: What information do you use? Where from? How do you access it? What do you do with it? Could you use more? What would you like? How would you like it? What would you do with it?<br>*Local data*: Do you use Monash Health data? How? What for? Do you use external data? What? How? What for? Could you use it? How?<br>*General discussion*: How could we get wider feedback? Should we survey, etc? Should this be driven top down or bottom up? Would you be interested in piloting something?<br>**Data collection:** Structured interviews were conducted using the interview schedule above; one CCE staff member attended and took notes.<br>**Analysis:** Responses were collated and added to findings from the other sources which were then analysed thematically by content analysis.<br>**Response rate:** All 6 informants participated.<br>**Representativeness of sample:** Interviewees represented senior decision-makers from a range of contexts

Table B

How are decisions about resource allocation currently made at Monash Health?

What factors influence decision-making for resource allocation?

What knowledge or experience of disinvestment exists within Monash Health?

Reproduced with permission from SHARE Paper 3 [4]

Structured interviews with staff authorised to make decisions on behalf of the organisation

Aim: To identify and document current processes for making, implementing and evaluating decisions and the factors that influence them.

Inclusion criteria: Staff and consumers authorised to make decisions regarding resource allocation for health technologies and clinical practices at organisation-wide level in group or individual settings.

Sampling: Purposive and snowball sampling was used.

- Twenty-two committees were initially identified from a governance structure diagram. A further 20 were identified through a snowballing method by asking participants in the subsequent interview process, senior managers and Quality Unit staff if they were aware of others. Fourteen of the 42 potential committees met the inclusion criteria (Capital Expenditure, Falls Prevention, Information Systems Governance, Joint Program Quality and Safety, Medication Safety, Operating Suite Product Evaluation, Nurse Standardisation of Practice, Resuscitation, Skin Integrity and Pressure Ulcer, Sterilising Services, Technology and Clinical Practice, Therapeutics and Transfusion Committees and the Executive Management Team).
- Approved Purchasing Units (APUs) have delegated authority from the Board to commit the organisation to a legal and/or financial obligation such as issuing a purchase order or signing a contract. Of the nine APUs, two had been included in the group decision-making committees (Capital Expenditure Committee and Executive Management Team) and five others met the inclusion criteria (Pharmacy, Health Technology Services, Equipment Services, Procurement and Clinical Purchasing, and Materials Management).
- Clinical managers from one clinical program selected for its high use of health technologies were identified from the program's intranet page. Individuals were selected purposively to represent all levels within the program's decision-making hierarchy; medical and surgical sub-specialties, nursing and quality management; and a range of campuses.

Approach: Personalised email invitations from the project team were sent to the Chair, Executive Sponsor and/or Secretary of 14 committees, managers of 5 APUs and 9 managers from the selected clinical program. Approval from the Nursing and Medical Program Directors was sought before approaching individuals from the selected program.

Interview schedule: Questions were based on a theoretical framework [4] and included details of the characteristics of the external environment; organisation; potential adopters; decisions; implementation strategies; barriers and enablers; degree of implementation; degree of practice change; patient, practitioner, system and economic outcomes; and respondents reflections on the current system. They were piloted with one committee and refined before being used in subsequent interviews. The full interview schedule is available [4].

Data collection: Interviews were approximately 1 hour long and were conducted in the interviewee's office or suitable meeting room. Interviews were not taped or transcribed but detailed notes were taken. Two CCE staff members attended, one as interviewer and one as note taker.

Respondent validation: Drafts were sent to the interviewees for clarification, comment and/or amendment as required.

Analysis: Final interview notes were collated and organised in MS Word and Excel using the elements of the theoretical framework. Emergent themes were identified by framework analysis.

Response rate: Representatives of 13 of the 14 committees, all 5 APU managers and 9 clinical managers participated. One committee Chair did not respond to the invitation for interview; due to lack of time no representative of this committee was interviewed. A surgical sub-specialty department head was unable to attend their interview and was replaced by a medical sub-specialty department head who was available at short notice.

Representativeness of sample: Almost all eligible committees and all eligible APUs were represented. The clinical managers represented Program Directors, Department Heads, Unit/Ward Managers and ancillary services; medical (n=4), nursing (n=4) and quality management (n=1) staff; in a range of sub-specialties across multiple campuses.

Structured interviews with staff members with experience in disinvestment projects

Aim: To learn from previous experiences of disinvestment at Monash Health.

Inclusion criteria: Staff who had undertaken projects to remove, reduce or restrict current practices (the term 'disinvestment' was not used in Monash Health projects).

Sampling: Purposive and snowball sampling was used. Relevant projects were initially identified by members of the SHARE Steering Committee and interviewees in the committee review process noted above. A snowballing method was employed by asking participating project representatives if they knew of any other relevant projects. Nineteen potential projects were identified, 13 met the inclusion criteria.

Approach: Personalised email invitations from the project team were sent to project managers of 13 relevant projects. Project managers or Department/Unit Heads were sought as key contacts; however a

representative of the project team was accepted when a senior staff member was unavailable.<br>**Interview schedule:** Questions were designed to explore project governance, use of routinely-collected hospital data, other local data and research evidence in the development and implementation of projects; barriers and enablers to successful project implementation; what staff would do again and what they would do differently. The full interview schedule is available in Table C.<br>**Data collection:** Interviews were approximately 1 hour long and were conducted in the interviewee's office or suitable meeting room. Interviews were not taped or transcribed but detailed notes were taken. Two CCE staff members attended, one as interviewer and one as note taker.<br>**Respondent validation:** Drafts were sent to the interviewees for clarification, comment and/or amendment as required.<br>**Analysis:** Final interview notes were collated and organised in MS Word and Excel using the elements of the theoretical framework noted above. Emergent themes were identified by framework analysis.<br>**Response rate:** Representatives of 10 projects participated based on interviewee's and interviewer's availability<br>**Representativeness of sample:** The process was designed to be illustrative and did not seek to comprehensively identify all projects. A number of project topics across a range of clinical areas were included.
**Structured workshops with the SHARE Steering Committee**
**Aim:** Workshop 1: To identify the need for change. Workshop 2: To develop a proposal for change<br>Additional details as above in Table A
**Structured workshop with clinical decision-makers from a large diagnostic service**
**Aim:** To capture the actual process of capital equipment purchasing and identify how an ideal process for this decision-making might differ from current practice.<br>**Inclusion criteria:** Clinical managers involved in decisions regarding purchase or new or replacement equipment.<br>**Sampling:** Purposive sampling was used. A large multi-campus diagnostic service was selected based on their use of equipment and the interest in the project expressed by the Director.<br>**Approach:** The Director and Research Director of the department identified 18 suitable participants representing all health professional groups, all campuses and most units within the service. Personalised email invitations were sent by the Executive Director of Medical Services and Quality.<br>**Design:** An experienced facilitator from CCE who had no involvement in the SHARE project developed and delivered the workshop. A presentation on the background of the project and its relevance to the workshop was made by a SHARE project team member. Two other project team members were present to assist with logistics and note taking. The session was run over 1½ hours in the departmental seminar room. Five domains were identified a priori: how do we get an idea; what is the process (application, approval, feedback, who, timing); is it a good idea; is it the best idea; and monitoring and evaluation.<br>**Data collection:** Using a nominal group technique, participants were asked to describe the ideal process for purchasing large capital equipment. Responses were collected on 'sticky-notes'. This method was repeated to identify gaps in the current process and included prioritisation of key areas for improvement.<br>**Respondent validation:** A workshop report was provided to participants for comment.<br>**Analysis:** Responses on the 'sticky notes' and additional workshop notes were collated and organised in MS Word and Excel using the domains identified a priori. Emergent themes were identified by framework analysis.<br>**Response rate:** 17 of the 18 invitees attended. An additional staff member from a clinical area not represented on the invitation list was included at the commencement of the workshop.<br>**Representativeness of sample:** Participants represented all campuses, sub-specialties and health professionals (medicine, nursing, allied health, technical, quality improvement, business management, research) within the department.
**Document analysis**
**Aim:** To provide evidence for the stated positions and methods of administration of decision-making systems and processes for resource allocation at Monash Health and the state health department.<br>**Inclusion criteria:** Documents that guided decision-making or implementation of resource allocation decisions<br>**Identification:** Documents were identified by key informants and searches within the Monash Health Policy and Procedure database.<br>**Documents included:** 1) State government: Victorian Government Purchasing Guidelines, Medical Equipment Asset Management Framework, Targeted Equipment Replacement Program and Health Purchasing Victoria Product Management Guidelines. 2) Monash Health: Purchasing Policy, Purchasing Policy Guidelines, Authority Delegation Schedule, Code of Conduct, Conflict of Interest Protocol, Guidelines for management of Gifts and Benefits, Terms of Reference for committees that make resource allocation decisions, Application forms, Business case templates, Requisition forms and checklists.<br>**Data collection:** Documents were retrieved or sourced online. Data were extracted based on the theoretical framework noted above.<br>**Analysis:** Findings were collated and organised in MS Word and Excel using the elements of the theoretical framework.

Table C

How can consumer values and preferences be integrated into organisation-wide decision-making processes?

Reproduced with permission from SHARE Paper 4 [5]

**Literature review**
**Aim:** To find evidence-based strategies that are effective in engaging consumers for health service organisation-wide decision-making processes. To find tools that can enable the implementation of these consumer engagement strategies. To find examples from other health services that employ evidence-based consumer engagement for organisation-wide decision-making processes.<br>**Protocol:** A two-step systematic review protocol was developed. The first step was to identify existing synthesised evidence and appraise it for quality and applicability; if no suitable publications were identified then a search of the primary research literature would follow. Relevant high-quality synthesised evidence in the form of guidance documents for consumer engagement were identified in the first step hence no further searches were undertaken. The full protocol is available [5]<br>**Search terms:** Search string for websites consisted of a combination of a 'consumer' term and an 'engagement' term. Where website search engines could not support truncation all terms were entered in full. Consumer terms: Consumer, Consumers, Community, Communities, Citizen, Citizens, Patient, Patients, Public. Engagement terms: Engagement, Engaging, Engage, Participation, Participating, Participate, Involvement, Involving, Consultation, Consulting, Deliberating, Deliberation, Deliberate, Input<br>**Sources:** All EBM (including Cochrane Database of Systematic Reviews, DARE, CENTRAL, and ACP Journal Club), Medline(R) 1950 to present with daily updates and Medline(R) in-process and other non-indexed citations, CINAHL, and EMBASE. Additional searches were undertaken in Guidelines websites (n=8), Databases and search engines (n=4), Government and consumer health organisation websites (n=7). Details of these websites are in the full protocol [5]. Checking of reference lists of articles, reports and links on websites was also undertaken.<br>**Selection criteria:** Detailed inclusion and exclusion criteria based on Population, Intervention/indicator, Comparison/control, Outcomes, Setting, Study design, Language and Time period were established a priori and applied by a single reviewer. Details of selection criteria are in the full protocol [5].<br>**Data Collection and Analysis:** All quality appraisals were conducted by a single reviewer in consultation with colleagues as required using criteria appropriate to the study design.<br>**Synthesis:** Findings were summarised by emergent themes and presented in detailed reports used for project decision-making and planning. Definitions, concepts related to consumer engagement for resource allocation in the local healthcare context and relationships between these concepts were extracted and collated with findings from the other sources and developed into a framework.<br>**Search results:** Eleven documents met the inclusion criteria and the requirement of step 1 as high quality synthesised evidence. These included systematic reviews, frameworks, toolkits and guidance for consumer engagement.
**Semi-structured workshops with SHARE Consumer Working Group**
**Aim:** To identify potential opportunities and methods for consumer participation and sources of consumer information.<br>**Inclusion criteria: C**onsumer representatives with experience in organisation-wide decision-making related to resource allocation.<br>**Sampling:** Convenience sampling was used. Three consumer representatives meeting the inclusion criteria were known to the project team (as members of committees overseeing introduction of new TCPs and development of policies and procedures), two of them were on the SHARE Steering Committee.<br>**Approach:** The three representatives were approached personally and invited to participate.<br>**Design:** Workshop 1 addressed the question: How can we capture consumer perspectives and include in decisions related to organisation-wide systems and processes? Prompts for discussion included<br>Methods of involvement: Who? How? Use of research literature and local/other data: What? Where? How? Who else to talk to? and Things we haven't thought of? Workshop 2 considered and refined the findings of Workshop 1 and added further detail.<br>**Data collection:** Group discussions were held at meetings convened for this purpose. Project team members took notes.<br>**Respondent validation:** Drafts were sent to the interviewees for clarification, comment and/or amendment as required.<br>**Analysis:** Responses were collated and added to findings from the other sources which were then analysed thematically by content analysis. Responses were summarised by emergent themes and presented in detailed reports used for project decision-making and planning. Concepts related to consumer engagement for resource allocation in the local healthcare context and relationships between these concepts were extracted and collated with findings from the other sources and developed into a framework.<br>**Response rate:** All members of the Consumer Working Group participated in both workshops.<br>**Representativeness of sample:** The consumer representatives were experienced in health service decision-making and familiar with organisational systems and processes.
**Semi-structured interviews with staff responsible for consumer-related activities**

**Aim:** To identify consumer-related activities within the organisation<br>**Inclusion criteria:** Staff with responsibility for consumer-related activities<br>**Approach:** Invitations for interview were sent to the Monash Health Quality Manager and Consumer Engagement Manager.<br>**Interview schedule:** What consumer-related activities occur within the organisation? What are your thoughts on findings from Consumer Working Group and interviews with Monash Health staff? Who else to talk to? Things we haven't thought of?<br>**Data collection:** Individual interviews were held at meetings convened for this purpose in the interviewee's office. One member of the project team was both interviewer and note taker.<br>**Respondent validation:** Drafts were sent to the interviewees for clarification, comment and/or amendment as required.<br>**Analysis: Responses were collated and added to findings from the other sources which were then analysed thematically by content analysis.** Responses were summarised by emergent themes and presented in detailed reports used for project decision-making and planning. Concepts related to consumer engagement for resource allocation in the local healthcare context and relationships between these concepts were extracted and collated with findings from the other sources and developed into a framework.<br>**Response rate:** Both invitees participated.<br>**Representativeness of sample:** The interviewees represented senior staff responsible for consumer participation in the organisation
**Structured interviews with decision-makers**
**Aim:** To ascertain current practice in consumer involvement in organisational decision-making and implementation and evaluation of change.<br>**Participants:** Monash Health staff authorised to make decisions on behalf of the organisation and project staff who had undertaken disinvestment-type projects (previously described in Table B).<br>**Interview schedule:** Questions regarding consumer participation in decision-making at Monash Health were a subset of the broader interviews which are reported in Table B.

Table D

What do Monash Health decision-makers need to enable access and utilisation of evidence in decision-making?

Reproduced with permission from SHARE Papers 7 and 8 [6, 7]

**Literature review**
**Aim:** To identify the information needs of decision-makers in local healthcare services<br>**Questions:** What are the information needs of clinicians and managers to support evidence-based decision making regarding the introduction or removal of technologies and clinical procedures?<br>How have assessments to determine these needs been conducted in the past?<br>**Sources:** Medline, CINAHL, EMBASE, LISA, LISTA and Google<br>**Medline Search** (adapted for other databases): (exp Needs Assessment/) AND (Information Dissemination/ or Information Services/ or Information Management/) limit to (english language and humans)<br>**Google Search:** (information OR evidence) AND (need OR assessment) AND (health OR nurs OR doctor OR med). Preferences were set to English language<br>**Inclusion criteria:** Articles describing information needs assessments in similar health service contexts examining how clinicians and managers make evidence-based decisions regarding the introduction or removal of technologies and clinical practices; articles published in English from 1996<br>**Exclusion criteria:** Information needs of students; continuing professional education needs; point of care decision-making needs; assessments of information needs in resource poor health settings<br>**Data Collection and Analysis:** Inclusion, exclusion and appraisal criteria were established a priori. Studies to be reviewed by one reviewer in consultation with colleagues when necessary. Critical appraisal relevant to study design to be conducted using standard CCE templates.<br>**Search results:** No studies were found to meet the inclusion criteria. The limitations of the very specific question and narrow selection criteria were acknowledged. Earlier broad searches resulted in unmanageable numbers of returned articles, however limiting the search returned none. Since the purpose of the review was to inform development of the support services, and not to be a systematic review providing a definitive answer for others, a decision was made to take a pragmatic, iterative approach by accessing relevant publications already known to the project team, pursuing articles from reference lists, etc.
**Structured interviews with Program, Department and Unit Heads**
**Aim:** To identify relevant issues and pilot draft questions for needs analysis survey<br>**Participants:** 9 managers including Program Directors, Medical Department Heads, Nurse Unit Managers and a Quality and Risk Manager from medical and surgical sub-specialties across a range of campuses<br>**Interview schedule:** Questions were designed to identify training and support needs for decision-making, implementation and evaluation and preferred formats for delivery. These were added to the schedule for interviews conducted to investigate organisational decision-making more broadly described in Table B above.
**Electronic survey of local decision-makers**
**Aim:** To identify the information needs of decision-makers at Monash Health to facilitate development of support services and gather baseline data for evaluation purposes<br>**Participants:** Staff who made decisions regarding resource allocation for technologies and clinical practices were eligible<br>**Design and content:** An electronic questionnaire was designed and delivered using SurveyMonkey [8]. Questions were developed to identify current use of evidence; confidence in searching for, accessing and appraising evidence; difficulties in using evidence and implementing evidence-based change; preferred content and format of bulletins disseminating research evidence; and preferred formats for education and training in these areas. Some questions were adapted from Taylor et al [9].<br>**Pretesting and piloting:** The survey was pre-tested with colleagues at a co-located research institute, piloted with the SHARE Steering Committee, and refined based on feedback from these groups<br>**Distribution:** An email with an embedded link to the survey was distributed to senior staff using the Monash Health 'All Managers' and 'Senior Medical Staff' email lists. Members of these lists were asked to forward the survey to others who made decisions about resource allocation but might not be on the list.<br>**Data collection:** Data were collected over a four week period from the time of distribution. No reminders were sent.<br>**Analysis:** Results were downloaded into Excel from the survey provider. Qualitative data from the three free text answers were copied into NVivo [10] where they were coded according to themes presented in Michie et al [11]. Data were reviewed by two investigators to ensure agreement of coding. Discrepancies were discussed with a third investigator until consensus was reached. There were insufficient categories in the Michie et al framework to address some of the organisational issues; additional sub-themes were created as required.<br>**Response rate:** 141 staff members responded. 118 were eligible to complete the survey having answered 'yes' to the screening question asking if they made decisions about resource allocation. 103 completed the entire survey. The response rate could not be calculated in the absence of denominator information; the total number of staff on the email lists and the number of additional staff to whom the survey was forwarded were unknown.<br>**Representativeness of sample:** All programs and service sites were represented in proportions consistent with the size of the program or campus. A range of professional disciplines were represented: nursing (28%), allied health (25%), medical (24%) and other (23%) including pharmacy, diagnostic services, corporate and clinical program management, and administration.

Table E

Program development

Structured workshops with SHARE Steering Committee

Aims: To review and refine draft proposals, frameworks and plans and make final decisions.

Participants: SHARE Steering Committee members including Executive Directors (Medical, Nursing, Support Services), Program Directors (Medical, Nursing, Allied Health, Pharmacy, Diagnostic Services), Committee chairs (Technology/Clinical Practice, Therapeutics, Human Research and Ethics, Clinical Ethics), Managers (Information Services, Clinical Information Services, Procurement, Biomedical Engineering, Research Services), Legal counsel and two Consumer representatives.

Design

- Provision of pre-reading materials and/or workshop presentation of background, issues to consider, draft proposals, etc
- Agenda including points for discussion and decisions required
- Decisions made by consensus
- Documentation of discussion, decisions and actions in minutes

Structured workshop with Community Advisory Committee (reproduced with permission from SHARE Paper 4 [5])

Aims: To identify current consumer engagement activities, barriers and enablers to effective participation in these situations and the needs of consumers in order to contribute effectively; to identify sources of consumer information and data and how these sources can be used to drive decision-making; and to seek feedback on a draft model for consumer engagement in generic health service decision-making

Inclusion criteria: The Community Advisory Committee is a legislated advisory body to the health service Board providing consumer, carer and community perspectives. This group provides a consultation service to health service staff engaging in consumer-related activities.

Approach: A request for consultation was completed on the required template. A workshop was held at a meeting convened for this purpose.

Design: The project team delivered a presentation that included the background and aims of SHARE, potential decision-making settings identified in earlier SHARE work, and findings from the literature review, interviews and consultation with staff and consumers regarding current and potential consumer participation in decision-making at Monash Health. This was followed by a structured discussion on the following topics:

- Committees and Working Parties: What would consumers need to contribute effectively? What are the barriers and enablers to effective participation? Other thoughts?
- Consultation: Are there particular areas we should focus on? What would consumers need to contribute effectively? What are the barriers and enablers to effective participation? Other thoughts?
- Using our consumer data: Sources we have identified. Are there others? Should there be others? How can we use this information to drive decision-making? How should consumers be involved in this process?
- Using our other data: Sources we have identified. How can we use this information to drive decision-making? How should consumers be involved in this process?
- Consumer literature: Suggestions. How can we use this information to drive decision-making? How should consumers be involved in this process?
- Is there anything else we have missed?

Data collection: Project staff took notes.

Analysis: Responses were collated and added to findings from the other sources which were then analysed thematically by content analysis.

Response rate: 6 of the 14 committee members attended the workshop

References

1. Harris C, Allen K, King R, Ramsey W, Kelly C, Thiagarajan M. Sustainability in Health care by Allocating Resources Effectively (SHARE) 2: Identifying opportunities for disinvestment in a local healthcare setting BMC health services research. 2017;(Details TBA).
2. Rumbold G, Allen K, Harris C. Disinvestment of technologies and clinical practices in health services: Conceptual and policy perspectives. Centre for Clinical Effectiveness, Southern Health 2008. Available from: http://arrow.monash.edu.au/hdl/1959.1/1218935. Accessed: October 2016
3. Harris C, Turner T, Wilkinson F. SEAchange: Guide to a pragmatic evidence-based approach to Sustainable, Effective and Appropriate change in health services. 2015. Available from: http://arrow.monash.edu.au/hdl/1959.1/1225377. Accessed: October 2016
4. Harris C, Allen K, Waller C, Brooke V. Sustainability in Health care by Allocating Resources Effectively (SHARE) 3: Examining how resource allocation decisions are made, implemented and evaluated in a local healthcare setting BMC health services research. 2017;(Details TBA).
5. Harris C, Ko H, Waller C, Sloss P, Williams P. Sustainability in Health care by Allocating Resources Effectively (SHARE) 4: Exploring opportunities and methods for consumer engagement in resource allocation in a local healthcare setting BMC health services research. 2017;(Details TBA).
6. Harris C, Allen K, Waller C, Dyer T, Brooke V, Garrubba M et al. Sustainability in Health care by Allocating Resources Effectively (SHARE) 7: Supporting staff in evidence-based decision-making, implementation and evaluation in a local healthcare setting BMC health services research. 2017;(Details TBA).
7. Harris C, Garrubba M, Melder A, Voutier C, Waller C, King R et al. Sustainability in Health care by Allocating Resources Effectively (SHARE) 8: Developing, implementing and evaluating an Evidence Dissemination Service in a local healthcare setting. BMC health services research. 2017;(Details TBA).
8. SurveyMonkey, . SurveyMonkey Inc., Palo Alto, California, USA. www.surveymonkey.com. Accessed March 2016.
9. Taylor R, Reeves B, Mears R, Keast J, Binns S, Ewings P et al. Development and validation of a questionnaire to evaluate the effectiveness of evidence-based practice teaching. Medical education. 2001;35(6):544-7.
10. NVivo qualitative data analysis software Version 8. QSR International Pty Ltd; 2008.
11. Michie S, Johnston M, Abraham C, Lawton R, Parker D, Walker A. Making psychological theory useful for implementing evidence based practice: a consensus approach. Quality & safety in health care. 2005;14(1):26-33. doi:10.1136/qshc.2004.011155.
